# Effectiveness of a virtual hospital model of care for patients with low back pain presenting to emergency departments (Back@Home)

**DOI:** 10.1038/s41746-026-02425-8

**Published:** 2026-02-27

**Authors:** Chathurani Sigera, Crystian B. Oliveira, Alla Melman, Min Jiat Teng, Danielle M. Coombs, Laurent Billot, Chris Gianacas, Alamgir Kabir, Eileen Rogan, Mona Marabani, Owen Hutchings, Chris G. Maher, Gustavo C. Machado, Back@Home Investigators, Ananthila Anandacoomarasamy, Bethan Richards, Chris Needs, Dane Chalkley, Ian Harris, James Edwards, Kirsten McCaffery, Michael Dinh, Miranda Shaw, Narcyz Ghinea, Qiang Li, Rachael Dodd, Rachelle Buchbinder

**Affiliations:** 1 School of Public Health, Faculty of Medicine and Health, The University of Sydney, Sydney, NSW Australia; 2 Institute for Musculoskeletal Health, Sydney Local Health District, Sydney, NSW Australia; 3 Sydney Virtual Hospital, Sydney Local Health District, Sydney, NSW Australia; 4 Biostatistics & Data Science, The George Institute for Global Health, University of New South Wales, Sydney, NSW Australia; 5 Royal Prince Alfred Hospital, Sydney Local Health District, Sydney, NSW Australia; 6 Rheumatology Department, Royal Prince Alfred Hospital, Sydney, NSW Australia; 7 Emergency Department, Royal Prince Alfred Hospital, Sydney, NSW Australia; 8 School of Clinical Medicine, Faculty of Medicine and Health, University of New South Wales, Sydney, NSW Australia; 9 Ingham Institute for Applied Medical Research, South Western Sydney Clinical School , University of New South Wales, Sydney, NSW Australia; 10 Sydney Health Literacy Lab, Sydney School of Public Health, Faculty of Medicine and Health, The University of Sydney, Sydney, NSW Australia; 11 RPA Green Light Institute for Emergency Care, Sydney Local Health District, Sydney, NSW Australia; 12 The Daffodil Centre, The University of Sydney and Cancer Council NSW, Sydney, NSW Australia; 13 Musculoskeletal Health and Wiser Health Care Units, School of Public Health and Preventive Medicine, Monash University, Melbourne, VIC Australia

**Keywords:** Diseases, Health care, Medical research

## Abstract

This study evaluated the effectiveness of a virtual hospital model of care (Back@Home) for low back pain (LBP). We conducted an observational study across four hospitals in the Sydney Local Health District, Australia. We included patients aged ≥16 years presenting to the ED with a primary diagnosis of non-specific or radicular (non-serious) LBP. We performed logistic or linear mixed models and multivariate linear regression analyses. From January 2017 to January 2025, 18,851 patients presented to the ED with non-serious LBP. The Back@Home model of care did not significantly affect admissions to inpatient or short-stay units (OR: 1.19, 95% CI: 0.46 to 3.06). However, it reduced ED re-presentations within 30 days (OR: 0.59, 95% CI: 0.42 to 0.84) by 41%. Patients who received virtual hospital care reported less pain (β: -1.26, 95% CI: -2.22 to -0.30), better physical function (β: 4.81, 95% CI: 2.36 to 7.27) and similar satisfaction (β: 0.14; 95% CI: -0.72 to 1.01) with care compared to those receiving inpatient care. Back@Home did not reduce admissions for LBP, but the model of care resulted in fewer ED re-presentations. Although causal inference cannot be made, virtual care may deliver health outcomes comparable to, or better than, traditional care.

## Introduction

Low back pain imposes an enormous burden on emergency departments (EDs) and inpatient services. In the United States, it accounts for approximately 13% of hospital admissions^1^. In England, low back pain was responsible for 365,327 ED presentations and 140,392 hospital admissions in the 2023–24 period^2^. In Australia, low back pain was the 5th most common reason for ED visits in 2023–2024, with one third of the patients subsequently admitted to hospital^3^—costing an average of approximately $15,000 per admission^4^. Another concern is that 8% of the patients with low back pain re-present to the ED within 12 months in Australia^5^ and 14% in the United States^6^. These re-presentations and admissions add to healthcare costs and strain ED capacity. Many ED re-presentations and hospital admissions for low back pain may be avoidable, as these patients are typically discharged with a non-serious low back pain diagnosis^5,7^.

Virtual care and community-based models, including home-based and outpatient services, have been proposed to reduce avoidable ED presentations and hospitalisations. A systematic review found that Hospital in the Home programmes facilitated early discharge, and admission avoidance models can reduce hospital length of stay and readmission rates, while achieving similar health outcomes to usual inpatient care^8^. A previous randomised trial found that a hybrid model (including home visits and virtual care) reduced healthcare costs and readmissions of patients with acute conditions (e.g., pneumonia)^9^. Despite these promising findings, none of these studies has yet tested the effectiveness of virtual hospital care models for patients with low back pain presenting to the ED.

This study evaluated the effectiveness of a virtual hospital model of care for low back pain (Back@Home) in reducing hospital admissions and re-presentations to the ED.

## Results

### Health service outcomes

From January 2017 to January 2025, there were 18,851 ED presentations for low back pain to the three EDs – 14,554 prior to, and 4297 following the introduction of the Back@Home model of care. The characteristics of the low back pain presentations were similar before and after we introduced the model (Table 1). In total, 260 patients received Back@Home service with a median length of stay of six days (IQR 3–11). Most patients had at least one consultation with a physician (86%) or a physiotherapist (92%).

**Table 1:** Characteristics of the low back pain presentations

Characteristic	Overall	Pre-implementation	Post-implementation
	*n* = 18,851	*n* = 14,554	*n* = 4297
Gender, *n* (%)			
Male	9,284 (49.2%)	7,194 (49.4%)	2,090 (48.6%)
Female	9,563 (50.7%)	7,357 (50.5%)	2,206 (51.3%)
Indeterminate	4 (0.0%)	3 (0.0%)	1 (0.0%)
Age, years; mean (SD)	51.3 (19.6)	51.1 (19.6)	51.9 (19.8)
Preferred language, *n* (%)			
Non-English	4,406 (23.4%)	3,399 (23.4%)	1,007 (23.4%)
English	14,442 (76.6%)	11,152 (76.6%)	3,290 (76.6%)
Mode of arrival, *n* (%)			
State Ambulance Vehicle	5,593 (29.8%)	4,400 (30.2%)	1,193 (28.3%)
Community/Public Transport	796 (4.2%)	667 (4.6%)	129 (3.1%)
Private car	11,131 (59.3%)	8,486 (58.3%)	2,645 (62.8%)
No transport (walked in)	1,171 (6.2%)	936 (6.4%)	235 (5.6%)
Other	73 (0.4%)	65 (0.4%)	8 (0.2%)

*SD* standard deviation.

Table 2 details the effects of the Back@Home model of care on health service outcomes. The introduction of Back@Home did not significantly change admissions to ED short stay or inpatient units (OR: 1.19, 95% CI: 0.46 to 3.06), nor in admissions to inpatient units alone (OR: 1.03, 95% CI: 0.80 to 1.33). ED re-presentations within 30 days significantly reduced (OR: 0.59, 95% CI: 0.42 to 0.84), indicating a 41% reduction in the likelihood of returning to the ED following Back@Home implementation. We found no significant differences in length of stay for patients admitted to ED short stay (*β*: 17.7 min, 95% CI: −59.29 to 94.63) or inpatient units (β: −0.12 days, 95% CI: −1.01 to 0.77).

**Table 2:** Health service outcomes

	Pre-implementation	Post-implementation	OR or *β* (95% CI)^a^
	n = 14,554	*n* = 4297	
Admitted to inpatient or ED short stay unit	3009 (20.7%)	885 (20.6%)	1.19 (0.46, 3.06)
Admitted to inpatient unit only	1404 (9.7%)	359 (8.4%)	1.03 (0.80, 1.33)
Re-presented to ED within 30 days	1152 (7.9%)	280 (6.5%)	**0.59 (0.42, 0.84)**
Length of ED short stay unit stay, mins	361.3 (351.8)	381.3 (353.4)	17.67 (−59.29, 94.63)
Length of inpatient stay, days	6.5 (8.1)	6.7 (7.5)	−0.12 (−1.01, 0.77)

Data are n (%) or mean (SD) unless otherwise specified.

^a^ Adjusted for seasonality, secular trend and COVID-19 periods.

Bold indicates *p* < 0.05.

Supplementary Table 4 describes the results of the sensitivity analysis per hospital site. After we introduced the model, overall admissions reduced significantly in one hospital (OR: 0.72, 95% CI: 0.57 to 0.91) but increased in the other two hospitals (OR: 1.35, 95% CI: 1.10 to 1.67; OR: 1.37, 95% CI: 1.03 to 1.83). We found no significant differences in admissions to inpatient units alone across the three hospitals, suggesting that changes in overall admissions were driven by variation in ED short stay admissions. ED re-presentations within 30 days reduced in two hospitals (OR: 0.48, 95% CI: 0.23 to 1.00; OR: 0.59, 95% CI: 0.36 to 0.97) and showed no difference in the third hospital (OR: 1.16, 95% CI: 0.62 to 2.17). Length of stay did not differ across hospitals.

### Patient-reported outcomes

Between December 2021 and May 2023, we invited a non-random subsample of 107 patients (mean age 54.6 years [SD: 18.7]; 53% female) who met Back@Home referral criteria and received traditional inpatient care to complete patient outcome surveys, with 53 (49%) completing all measures at 2-week follow-up. From February 2023 to January 2025, we invited all 260 patients who received Back@Home care (mean age 47.1 years [SD: 16.5]; 46% female) to complete the same survey, with 106 (41%) completing all measures at 2-week follow-up. Patients receiving Back@Home care reported significantly less pain (β: −1.26, 95% CI: −2.22 to −0.30) and better physical function (*β*: 4.81, 95% CI: 2.36 to 7.27), while reporting similar satisfaction with care (β: 0.14; 95% CI: −0.72 to 1.01) when compared to patients receiving usual inpatient care (Table 3).

**Table 3:** Patient-reported outcomes at 2-week follow-up

	Usual inpatient care (*n* = 53)	Virtual hospital care (*n* = 106)	*β* (95% CI)^a^
Pain intensity (0-10)	5.6 (2.6)	4.2 (2.5)	**−1.26 (−2.22 to −0.30)**
Physical function (0-30)	14.6 (7.3)	20.7 (6.5)	**4.81 (2.36 to 7.27)**
Satisfaction with care (0-10)	7.6 (2.6)	7.9 (2.3)	0.14 (**−**0.72 to 1.01)

Data are mean (SD) unless otherwise specified.

^a^Adjusted for age, sex, triage category, mode of arrival and socio-economic status.

Bold indicates *p* < 0.05.

Data are missing for triage category (*n* = 1), mode of arrival (*n* = 1) and socio-economic status (*n* = 4).

## Discussion

Overall admissions did not reduce, after introducing the Back@Home model of care; however, ED re-presentations within 30 days dropped significantly, with patients being 41% less likely to re-present. Subgroup analyses showed that the impact on admissions and ED re-presentations varied across the three hospitals. We did not find any differences in length of stay. Although the observational nature of the data limits causal inference, patients receiving Back@Home care consistently reported less pain, better physical function, and satisfaction with care levels comparable to those receiving usual hospital care.

Our study provides evidence using routinely collected data from the electronic medical records of 18,851 presentations with low back pain across an 8-year period, which enhances its external validity. However, there are some limitations. Because our study was observational, we cannot draw definitive conclusions or make causal inferences, as it was not a randomised controlled trial. Although we adjusted our analysis for secular trend, seasonality and if the presentation occurred during a COVID-19 restriction period, we cannot exclude the possibility of additional confounding factors. We established the Back@Home programme in three EDs and a virtual hospital of a single Local Health District (a network of public hospitals and health services) in New South Wales, which may restrict the generalisability of our findings. Clinicians and hospital coders performed the diagnostic coding as part of standard practice, which could have influenced the number of presentations over time due to variations in coding. In addition, the use of diagnosis codes to select the eligible presentations cannot exclude the possibility of having included patients with serious radicular pain requiring surgery. Only half of the patients we invited completed the patient-reported outcome measure surveys. Therefore, these findings should be interpreted with caution because we cannot exclude the possibility of selection and attrition bias. Finally, although we attempted to recruit patients with similar characteristics and adjusted the analysis for age, sex, triage category, mode of arrival, and socio-economic status, we did not collect extensive baseline measures and cannot exclude the possibility that the groups differ on other factors.

This study is the first to investigate the effectiveness of a virtual hospital model of care for low back pain. Our findings align with those from previous studies investigating the effects of virtual care. For instance, a recent meta-analysis revealed that virtual ward models significantly reduce readmissions, with patients reporting high satisfaction with care^10^. While some studies investigated the effects of virtual models of care in specific populations (e.g., pneumonia and chronic obstructive pulmonary disease)^10^, none have investigated the effects of virtual wards for musculoskeletal conditions on health service outcomes. Our study also provides preliminary evidence that patients receiving virtual care reported better symptom outcomes than those receiving traditional inpatient care. These findings align with a previous review showing that telehealth programmes can improve function and reduce pain in people with knee or hip osteoarthritis when compared to usual care^11^.

The introduction of the Back@Home model of care in the Sydney Local Health District showed no clear evidence of reducing overall admissions, nor did it significantly impact the length of stay for admitted patients. This may reflect the age eligibility criterion of the current model of care (i.e. <70 years) and the relatively short post-implementation evaluation period. Future evaluations considering older patients and longer follow-up periods may produce different findings and improve the precision of our estimates. Importantly, our study demonstrated that the Back@Home model of care was associated with a significant reduction in ED re-presentations. Reducing avoidable ED re-presentations is a national health priority in Australia^12^, as it supports better health outcomes, enhances patient safety, and improves health system efficiency by reducing healthcare costs and ED crowding. Our subgroup analysis revealed varied effects across hospitals, which may reflect differences in patient demographics, case-mix, and local clinical priorities, as well as the extent and fidelity of Back@Home model implementation across sites. These results, alongside clinicians’ feedback on refining and broadening patient selection criteria^12^, can inform further adaptations to standardise the impact of the Back@Home model of care across sites.

Although our study showed promising results of the new virtual hospital care model for low back pain, further research is warranted to build on these findings. We considered older people aged over 70 years with low back pain on a case-by-case basis to receive the Back@Home service, as there is still uncertainty about the safety and effectiveness of virtual care models in this population. Avoiding hospital admission in this population is crucial, as they face a higher risk of in-hospital complications such as acquired infections and falls^13^. Future trials should investigate the effectiveness and safety of a revised Back@Home model of care designed to cater to the needs of older people with low back pain. We did not collect data on transfers to external rehabilitation or residential care facilities, which would help better understand subsequent health-care utilisation and between-hospital variations identified in the sensitivity analysis. Future studies should investigate if subsequent health care utilisation (e.g., rehabilitation) could potentially influence re-presentation and readmission rates. Australia’s first virtual hospital, Sydney Virtual Hospital, launched in early 2020 in the Sydney Local Health District, which cared for nearly 5000 patients in 2023–24, and delivered Back@Home. Additional studies should investigate the feasibility and viability of a virtual hospital service for low back pain across other jurisdictions in Australia and other countries. While the cost-effectiveness of implementing Back@Home remains unclear, a 41% reduction in re-presentations to ED is potentially a very significant financial benefit to healthcare organisations. We are currently investigating this in a separate study.

The Back@Home model of care did not lead to significant reductions in overall hospital admissions (despite differences between sites), but it was associated with a meaningful reduction in ED re-presentations – a key national health priority in Australia. Patients receiving Back@Home care reported outcomes that were comparable to, and sometimes better than, those receiving usual hospital care. Although causality cannot be inferred, these preliminary findings suggest that virtual care can be delivered effectively outside the hospital. Our findings position Back@Home as a promising and scalable approach to improve service delivery and patient outcomes for people with low back pain presenting to ED. We need to further evaluate the effects of the model in other populations, such as older adults and patients requiring neurosurgical review, as well as testing the model rigorously in randomised controlled trials.

## Methods

### Study design and participants

We conducted an observational study in three Sydney Local Health District EDs, which collectively manage more than 179,000 ED attendances annually^14^, and a virtual hospital. We followed the Strengthening the Reporting of Observational Studies in Epidemiology (STROBE) Statement^15^ for observational studies (Supplementary Table 1). We published the study protocol elsewhere^16^. We obtained the ethical approval from the Sydney Local Health District Human Research Ethics Committee (RPAH Zone) for study protocols X21-0278 & 2021/ETH10967, and X21-0094 & 2021/ETH00591. The Sydney Local Health District Human Research Ethics Committee waived the requirement for individual informed consent, as the study involved analysis of routinely collected de-identified clinical data. We obtained informed verbal consent from participants for the collection of patient-reported outcomes.

We included presentations of patients 16 years or older who had a diagnosis of non-specific or radicular (non-serious) low back pain at ED discharge in this study. We identified eligible ED presentations from the electronic medical record system using a list of diagnosis codes from the Systematised Nomenclature of Medicine Clinical Terms—Australian Version (SNOMED CT-AU) (Supplementary Table 2). We only included patients with a hospital discharge diagnosis of non-serious low back pain based on the International Classification of Diseases, 10th Revision, Australian Modification (ICD-10-AM) (Supplementary Table 3). We excluded ED presentations and hospital admissions with diagnosis codes indicating serious spinal pathologies (e.g., vertebral fracture, spinal abscess, cauda equina syndrome, or malignancy).

### Intervention

We used the Back@Home model of care as the intervention for patients with low back pain. The Sydney Virtual Hospital Working Group, consisting of health managers, back pain researchers, rheumatologists, general practitioners, ED physicians, physiotherapists, consumers and Aboriginal Health representative developed the model of care. We introduced the Back@Home model of care on 13 February 2023 at the first site, with subsequent rollouts on 15 May 2023 and 29 May 2023 at two additional sites. This study focuses on the effectiveness of the Back@Home model of care; therefore, we have described the implementation strategies used to establish the service (e.g., educational meetings, local opinion leaders) in detail elsewhere^16,17^. We will report the results related to implementation outcomes (e.g., reach, adoption, implementation, and maintenance) in a separate publication.

The Back@Home model of care is a service operated by Sydney Virtual Hospital in the Sydney Local Health District. Patients eligible for the programme were those who would typically require hospital admission for ongoing care but were considered clinically safe for discharge, as well as those whom clinicians believed required additional follow-up to prevent an unplanned ED re-presentation. ED medical staff in consultation with virtual hospital consultants referred the eligible patients to the Back@Home service. We considered the patients eligible if they presented to the ED with a provisional diagnosis of non-serious low back pain, were able to mobilise a short distance (with or without a walking aid) and were deemed clinically safe for discharge home. We also considered that patients already admitted to the inpatient wards could be referred to the Back@Home service to facilitate earlier discharge.

The service is a hybrid model of care, including virtual support and home visits, if needed. Patients admitted to the Back@Home service received telehealth consultations with general practitioners and physiotherapists from Sydney Virtual Hospital via telephone calls, video consultations, a dedicated website, Short Message Services (SMS), or email. Patients had 24/7 access to Sydney Virtual Hospital support through a dedicated call centre with nursing staff. Care delivery also incorporated healthcare interpreters and educational materials on low back pain translated into the local community languages. We arranged home visits from physiotherapists when deemed necessary by the multidisciplinary team during regular clinical reviews. We used digital platforms, such as Physitrack mobile app to provide tailored exercise programmes. We delivered a three-day supply of FlexEze heat wraps directly to patients’ homes.

### Data collection and outcome measures

We collected data retrospectively from January 2017 to January 2025 from the hospitals’ electronic medical record systems to measure the effects of the Back@Home model of care on health service outcomes. We evaluated the proportion of patients admitted to either ED short stay or inpatient units following an ED presentation for low back pain as the primary outcome. As secondary outcomes, we evaluated admissions to inpatient units only, ED re-presentations within 30 days, and length of stay in ED short stay and inpatient units.

We prospectively collected patient-reported outcomes using REDCap for a sub-sample of patients who met Back@Home referral criteria and received traditional inpatient care prior to implementation (from December 2021 to May 2023) and for patients who received virtual hospital care through the Back@Home model of care (from February 2023 to January 2025). We measured pain intensity, physical function, and satisfaction with care at 2-week follow-up. We measured pain intensity using a 0–10 Numeric Pain Rating Scale, with higher scores indicating worse pain and physical function with PROMIS Physical Function Short Form (scale range 6–30), with higher scores reflecting better function. We measured global satisfaction with care on a 0–10 scale using a single question, with higher scores indicating higher satisfaction with the care received.

### Statistical analysis

We published the statistical analysis plan of this study on the Open Science Framework (https://osf.io/k287q). Because all participating hospitals implemented Back@Home and no concurrent unexposed control group was available, we used an analysis approach similar to interrupted time series with individual patient data. For health service outcomes, we compared the differences before and after implementing the Back@Home model of care based on the index date of patients’ presentations to the ED. We performed logistic or linear mixed-effects models with random intercepts, including fixed effects for the main exposure (pre versus post implementation), secular trend, seasonality, and whether the ED presentation occurred during a COVID-19 pandemic restriction period (i.e., February 1 to June 30, 2020; June 1 to December 1, 2021; and January 1 to May 1, 2022). We specified random effects for hospital site and the interaction between the main exposure and hospital site. For patient-reported outcomes at 2-week follow-up, we estimated the differences between the sub-sample of patients who received traditional inpatient care (before implementation) or Back@Home care using multivariate linear regression analyses (adjusted for age, sex, triage category, mode of arrival, and socio-economic status). We conducted all analyses using RStudio (R version 4.4.2) or SAS software version 8.3.

## Supplementary information

Supplementary information

## Data Availability

All data requests should be submitted to the corresponding author for consideration. Access to anonymised data may be granted following a review process.

## Code availability

The underlying code for this study is not publicly available but may be made available on reasonable request from the corresponding author.
