# Supplementary material for: Effectiveness of a virtual hospital model of care for patients with low back pain presenting to emergency departments (Back@Home)
Source: NPJ Digit Med. 2026 Feb 27;9:191. doi: 10.1038/s41746-026-02425-8 (PMC12948941; doi:10.1038/s41746-026-02425-8)
Supplement: Supplementary file 1 — Supplementary information [file 41746_2026_2425_MOESM1_ESM.pdf]

Supplementary Table 1: STROBE Statement—Checklist of items that should be included in reports of cohort studies

	Item No	Recommendation	Page No
**Title and abstract**	1	(*a*) Indicate the study's design with a commonly used term in the title or the abstract	1
		(*b*) Provide in the abstract an informative and balanced summary of what was done and what was found	2
**Introduction**			
Background/rationale	2	Explain the scientific background and rationale for the investigation being reported	3
Objectives	3	State specific objectives, including any prespecified hypotheses	3
**Methods**			
Study design	4	Present key elements of study design early in the paper	3
Setting	5	Describe the setting, locations, and relevant dates, including periods of recruitment, exposure, follow-up, and data collection	3-4
Participants	6	(a) Give the eligibility criteria, and the sources and methods of selection of participants. Describe methods of follow-up	3-4
		(b) For matched studies, give matching criteria and number of exposed and unexposed	5
Variables	7	Clearly define all outcomes, exposures, predictors, potential confounders, and effect modifiers. Give diagnostic criteria, if applicable	4-5
Data sources/ measurement	8*	For each variable of interest, give sources of data and details of methods of assessment (measurement). Describe comparability of assessment methods if there is more than one group	4-5
Bias	9	Describe any efforts to address potential sources of bias	5
Study size	10	Explain how the study size was arrived at	5
Quantitative variables	11	Explain how quantitative variables were handled in the analyses. If applicable, describe which groupings were chosen and why	5
Statistical methods	12	(a) Describe all statistical methods, including those used to control for confounding	5
		(b) Describe any methods used to examine subgroups and interactions	5
		(c) Explain how missing data were addressed	5
		(d) If applicable, explain how loss to follow-up was addressed	5
		(e) Describe any sensitivity analyses	5
**Results**			
Participants	13*	(a) Report numbers of individuals at each stage of study—eg numbers potentially eligible, examined for eligibility, confirmed eligible, included in the study, completing follow-up, and analysed	5
		(b) Give reasons for non-participation at each stage	-
		(c) Consider use of a flow diagram	-
Descriptive data	14*	(a) Give characteristics of study participants (eg demographic, clinical, social) and information on exposures and potential confounders	5
		(b) Indicate number of participants with missing data for each variable of interest	5-6
		(c) Summarise follow-up time (eg, average and total amount)	6
Outcome data	15*	Report numbers of outcome events or summary measures over time	5-6

Main results	16	(*a*) Give unadjusted estimates and, if applicable, confounder-adjusted estimates and their precision (eg, 95% confidence interval). Make clear which confounders were adjusted for and why they were included	5-6
		(b) Report category boundaries when continuous variables were categorized	5-6
		(c) If relevant, consider translating estimates of relative risk into absolute risk for a meaningful time period	-
Other analyses	17	Report other analyses done—eg analyses of subgroups and interactions, and sensitivity analyses	5-6
**Discussion**			
Key results	18	Summarise key results with reference to study objectives	6
Limitations	19	Discuss limitations of the study, taking into account sources of potential bias or imprecision. Discuss both direction and magnitude of any potential bias	6-7
Interpretation	20	Give a cautious overall interpretation of results considering objectives, limitations, multiplicity of analyses, results from similar studies, and other relevant evidence	6-8
Generalisability	21	Discuss the generalisability (external validity) of the study results	6-8
**Other information**			
Funding	22	Give the source of funding and the role of the funders for the present study and, if applicable, for the original study on which the present article is based	8

Supplementary Table 2: SNOMED CT-AU diagnosis codes related to LBP presentations

DESCRIPTION	CODES
**Low back pain with non-specific cause**	
Acute low back pain (finding)	278862001
Back pain complicating pregnancy (disorder)	91957002
Backache (finding)	161891005
Blunt injury to back (disorder)	424270008
Chronic back pain (finding)	134407002
Chronic low back pain (finding)	278860009
Coccyx sprain (disorder)	209571002
Complaining of low back pain (finding)	161894002
Degeneration of lumbar intervertebral disc (disorder)	26538006
Displacement of lumbar intervertebral disc without myelopathy (disorder)	20021007
Exacerbation of backache (finding)	135860001
Low back pain (finding)	279039007
Low back strain (disorder)	300956001
Lower back injury (disorder)	282766005
Lumbar spondylosis (disorder)	239880009
Lumbar sprain (disorder)	209565008
Mechanical low back pain (finding)	279040009
Pain in the coccyx (finding)	34789001
Sacral back pain (finding)	61486003
Spasm of back muscles (finding)	203095000
Sprain of ligament of lumbosacral joint (disorder)	209548004
Stiff back (finding)	249921008
Strain of back muscle (disorder)	262965006
Strain of tendon of back (disorder)	262975009
**Low back pain with neurological signs and symptoms**	
Acute back pain with sciatica (finding)	247366003
Acute sciatica (disorder)	307176005
Chronic sciatica (disorder)	307177001
Injury of lumbar nerve roots (disorder)	24300005
Injury of sciatic nerve (disorder)	86269002
Lumbago with sciatica (finding)	202794004
Lumbago-sciatica due to displacement of lumbar intervertebral disc (disorder)	46960006
Lumbar disc prolapse with radiculopathy (disorder)	202735001
Lumbar radiculopathy (disorder)	128196005
Sciatica (disorder)	23056005
Spinal stenosis of lumbar region (disorder)	18347007

SNOMED CT-AU, Systematized Nomenclature of Medicine Clinical Terms - Australian Version.

Supplementary Table 3: ICD-10 diagnosis codes related to non-serious LBP presentations

DESCRIPTION
**Low back pain with non-specific cause:**
M40.26 - Other and unspecified kyphosis, lumbar region
M41.96 - Unspecified scoliosis, lumbar region
M43.00 - Spondylolysis, multiple sites in spine
M43.04 - Spondylolysis, thoracic region
M43.05 - Spondylolysis, thoracolumbar region
M43.06 - Spondylolysis, lumbar region
M43.07 - Spondylolysis, lumbosacral region
M43.16 - Spondylolisthesis, lumbar region
M43.17 - Spondylolisthesis, lumbosacral region
M43.26 - Other fusion of spine, lumbar region
M43.86 - Other specified deforming dorsopathies, lumbar region
M47.84 - Other spondylosis, thoracic region
M47.85 - Other spondylosis, thoracolumbar region
M47.86 - Other spondylosis, lumbar region
M47.87 - Other spondylosis, lumbosacral region
M47.94 - Unspecified spondylosis, thoracic region
M47.95 - Unspecified spondylosis, thoracolumbar region
M47.96 - Unspecified spondylosis, lumbar region
M47.97 - Unspecified spondylosis, lumbosacral region
M53.86 - Other specified dorsopathies, lumbar region
M54.6 - Pain in thoracic spine
M54.5 - Low back pain
M54.80 - Other dorsalgia, multiple sites in spine
M54.84 - Other dorsalgia, thoracic region
M54.85 - Other dorsalgia, thoracolumbar region
M54.86 - Other dorsalgia, lumbar region
M54.87 - Other dorsalgia, lumbosacral region
M54.88 - Other dorsalgia, sacral and sacrococcygeal region
M54.89 - Other dorsalgia, site unspecified
M54.90 - Unspecified dorsalgia, multiple sites in spine
M54.94 - Unspecified dorsalgia, thoracic region
M54.95 - Unspecified dorsalgia, thoracolumbar region
M54.96 - Unspecified dorsalgia, lumbar region
M54.99 - Unspecified dorsalgia, site unspecified
S29.0 - Injury of muscle and tendon at thorax level
S30.0 - Contusion of lower back and pelvis
S30.80 - Other superficial injuries of abdomen, lower back and pelvis, unspecified
S30.81 - Other superficial injuries of abdomen, lower back and pelvis, abrasion
S30.88 - Other superficial injuries of abdomen, lower back and pelvis, other
S30.90 - Superficial injury of abdomen, lower back and pelvis, part unspecified, unspecified
S30.98 - Superficial injury of abdomen, lower back and pelvis, part unspecified, other

S33.51 - Sprain and strain of lumbosacral [joint] [ligament]
S33.6 - Sprain and strain of sacroiliac joint
S33.7 - Sprain and strain of other and unspecified parts of lumbar spine and pelvis
S39.0 - Injury of muscle and tendon of abdomen, lower back and pelvis
S39.8 - Other specified injuries of abdomen, lower back and pelvis
S39.9 - Unspecified injury of abdomen, lower back and pelvis

Low back pain with neurological signs and symptoms

G54.1 - Lumbosacral plexus disorders
G54.4 - Lumbosacral root disorders, not elsewhere classified
G54.9 - Nerve root and plexus disorder, unspecified
G55.0 - Nerve root and plexus compressions in neoplastic disease (C00-D48+)
G55.1 - Nerve root and plexus compressions in intervertebral disc disorders (M50-M51+)
G55.2 - Nerve root and plexus compressions in spondylosis (M47.-+)
G55.3 - Nerve root and plexus compressions in other dorsopathies (M45-M46+, M48.-+, M53-M54+)
G57.0 - Lesion of sciatic nerve
G57.1 - Meralgia paraesthetica
G58.8 - Other specified mononeuropathies
M47.14 - Other spondylosis with myelopathy, thoracic region
M47.26 - Other spondylosis with radiculopathy, lumbar region
M47.27 - Other spondylosis with radiculopathy, lumbosacral region
M48.00 - Spinal stenosis, multiple sites in spine
M48.05 - Spinal stenosis, thoracolumbar region
M48.06 - Spinal stenosis, lumbar region
M48.07 - Spinal stenosis, lumbosacral region
M48.08 - Spinal stenosis, sacral and sacrococcygeal region
M48.09 - Spinal stenosis, site unspecified
M51.1 - Lumbar and other intervertebral disc disorders with radiculopathy (G55.1*)
M51.2 - Other specified intervertebral disc displacement
M51.3 - Other specified intervertebral disc degeneration
M51.8 - Other specified intervertebral disc disorders
M54.15 - Radiculopathy, thoracolumbar region
M54.16 - Radiculopathy, lumbar region
M54.17 - Radiculopathy, lumbosacral region
M54.18 - Radiculopathy, sacral and sacrococcygeal region
M54.19 - Radiculopathy, site unspecified
M54.3 - Sciatica
M54.4 - Lumbago with sciatica
S33.0 - Traumatic rupture of lumbar intervertebral disc
S34.2 - Injury of nerve root of lumbar and sacral spine

Supplementary Table 4: Effects of the implementation of the virtual model of care (Back@Home) on health services outcomes in each site

Outcome	Site 1	Site 2	Site 3
Admitted to ED short-stay or inpatient unit	1.35 (1.10,1.67)	0.72 (0.57, 0.91)	1.37 (1.03, 1.83)
Admitted to inpatient unit	1.26 (0.94, 1.69)	0.96 (0.70, 1.32)	0.76 (0.47, 1.23)
Length of stay in ED short-stay unit	49.02 (-37.64, 135.69)	78.75 (-12.78, 170.27)	-76.60 (-231.87, 78.67)
Length of stay in inpatient units	0.01 (-2.1, 2.11)	0.76 (-1.66, 3.18)	-2.56 (-7.00, 1.88)
ED Representations within 30 days	0.48 (0.23,1.00)	1.16 (0.62, 2.17)	0.59 (0.36, 0.97)
